# The Action of Animal Poisons

**Published:** 1921-03-19

**Authors:** 


### A CHAPTER IN TROPICAL MEDICINE.

### The Action of Animal Poisons.

The appearance of the seventh edition of Manson's "Tropical Diseases"* provides the profession with an excellent record of advances during and since the war. There is hardly any part of this scientific field in which substantial progress has not been made. In those conditions of parasitic origin many life-histories have been further worked out and many completely new groups have come to light. But the technical knowledge now required by workers in these subjects is so great that the authors devote a series of appendices to special protozoölogy, medical zoology, and laboratory methods, and this is a most satisfactory procedure. It clears the main text of a mass of detail and leaves the bulk of the manual as highly instructive and entertaining reading to any traveller in the tropics, whether he be medical or not.

From this standpoint one might review in detail a host of highly interesting chapters, but, for its general interest, that dealing with snake venom and allied poisons claims due attention.

### The Venom of Snakes.

Most of us know that whilst vipers or adders are the only poisonous snakes found in Europe, the extremely deadly colubrine snakes (cobras, kraits, etc.) abound in Asia. Also that in the American Continent the most poisonous viperines of the family *Crotalinæ* are met with, these including rattlesnakes, copperheads, and mocassins as the most important. But North Africa can also show one species of cobra and two vipers, whilst the South of that continent has a different cobra, the puff adder and another viperine. Australia can claim a large variety of venomous colubrines, whilst the sea-snakes of the Pacific and Indian Oceans are, with one exception, all highly poisonous.

The size of the reptile has, in most cases, little definite relationship to the toxicity of its venom and also the lethal action on different species of animals is extremely variable. Another factor in forecasting the possible effect upon man is the quantity of poison actually carried in the snake's glands. This also varies greatly; and, although the toxicity per volume may be high, the quantity possibly injected at a bite is often the more important consideration. Therefore their relative danger to man does not run parallel to their zoological classification, and this fact probably accounts for many of the inconsistent results obtained in treatment of snakebite by antitoxin measures.

### Two Great Classes.

This treatment will be dealt with later, for, in the first place, it is necessary to realise what type of "poison" these snake venoms contain. Their action appears to depend on ferments and lysins, protein bodies capable of inactivating or breaking up living cells in the victim's

* "Manson's Tropical Diseases." Seventh edition. Revised and enlarged. Edited by Philip H. Manson-Bahr. Published by Cassell and Co., Ltd. Price 30s. net.

**A Chapter in Tropical Medicine—(*continued*).**

body. Ferments have been found which act upon both fibrin and protein; lysins which break up red blood cells, leucocytes, and nerve cells; agglutinins inactivating the free cells of the blood; and neurotoxins which temporarily destroy the function of nervous tissue, particularly the centres controlling respiration. It is particularly important that the neurotoxins predominate in colubrine poison, while those which act on the blood cells, and therefore on the circulatory system, are most characteristic of the viperine species.

To a large extent, then, it is possible to predict, the species being known, the effect of a snake-bite. The two great groups, colubrine and viperine, will serve as examples. In the case of cobra-bite, nervous paralysis is predominant. There is severe pain in the wound, but not until an hour later does the main effect appear, when the patient becomes dull, apathetic, and unable to stand. Nausea, vomiting, with paralysis of the tongue and larynx supervene, and gradually, the respiratory centre being involved, breathing ceases. Should the amount of toxin injected be such that the paralytic stage is survived, recovery is then remarkably rapid, and no sequelæ occur.

On the other hand, the effect of viperine toxin is widely different. Far greater local effect is produced, and the action being largely upon the blood itself, constitutional symptoms sometimes appear within fifteen minutes. Discoloration rapidly extends up the limb to the trunk, followed by general symptoms of blood poisoning, high temperature, prolonged restlessness, and delirium. The wound itself ultimately suppurates freely and may even become gangrenous before death occurs.

These descriptions suggest a high mortality rate. From most venomous snakes, however, it is not nearly so great as popularly supposed, and certainly not higher than about 30 per cent. The reptile seldom has the chance to inject a full dose of venom into a man. On the other hand, if given an unprotected skin surface and time enough the cobra can in one bite release ten times the fatal human dose.

Early Treatment.

In early treatment of any snake-bite, therefore, the possibility of these high doses, uncommon though they are, must be borne in mind. The first aim is to prevent absorption. A tight ligature round the limb is very effective in localising viperine poison, but has little effect upon the neuro-toxins of the cobra group. Amputation of the part is a drastic remedy, and in practice little better than the ligature. In any case, the next step is to destroy the poisons remaining in the region of the bite. This is always best effected by free incision and swabbing out with potassium permanganate, which destroys all toxins it comes across. Contrary to the popular idea, sucking of the wound is useless. Also alcohol and strychnine, so frequently recommended, are now known to have, except as stimulants, no effect whatever. Adrenalin may help to counteract the paralysis of the vaso-motor centres, but the rational treatment, if only it is available, is injection of a specific antiserum, or *antivenine*.

Calmette has made several attempts to produce a polyvalent serum efficient against all snake-bites, but so far there has been one serious difficulty. Only the antiserum specific to the snake concerned will be of use. It is impossible to immunise a domestic animal (the source of the serum) against more than one or two species of snakes, and even then the antivenines are relatively weak in their action compared, for example, with antidiphtheritic serum. Therefore the only practical method is to issue an antiserum against the most dangerous or commoner snakes of a district. These sera must be injected *intravenously* (100 c.c.) at the earliest possible moment, as they are only effective if given before the minimum lethal dose of venom has been actually absorbed into the system. Sooner or later a true polyvalent serum will probably be obtained, but the difficulties of injecting the necessary horses with snake venom are great, for intense abscess formation always occurs, and the whole process may last as long as a year and a-half before a satisfactory immunity is obtained.

Other Animal Poisons.

While on the subject of animal poisons it is well to remember some truths concerning species other than snakes. There is a prevalent belief that many lizards are poisonous. Actually only one, an inhabitant of Mexico, is certainly so, and there is one other doubtful instance, a jumping lizard of South-West Africa. Fish, however, are more frequent offenders. Especially among the Pacific and Indian coral reefs, one finds species which secrete a true venom from certain epithelial glands in the mouth. The teeth are not completely perforated, but the poison simply courses down a groove on their posterior surface. On man the effect is neuro-cardiac. A more common class of poisonous fish is that which carries spines capable of piercing the human skin. The poison lies within these spines and reaches the surface only when the points are broken off. Apart from its effect as a local irritant, this poison may produce marked tetanic symptoms and a cardiac depressant effect. Scorpion stings, although supposed to be highly dangerous, are not usually so to adults, although the venom is closely allied to that of snakes. Deaths have occurred among children, but an efficient antiserum can be prepared, and 5 c.c. of it produces both a prophylactic and a curative action. Nearly all spiders possess poison glands and produce a venom highly fatal to insects, but relatively few are dangerous to man. Their poison belongs to the lysin class, and is in some ways similar to but weaker than viperine toxin. At most they produce, in the healthy human, local inflammation, and if a considerable quantity is injected, mild jaundice and hæmoglobinuria, due to the destruction of a certain number of red blood cells. In all these instances the method of treatment is essentially the same. Potassium permanganate will destroy toxins locally if used with sufficient vigour, and alkalies or ammonia are generally efficacious in reducing the more irritant constituents of the venom.

The outline thus given is but an incomplete account of a highly important but often misunderstood section of tropical medicine. If the reader would follow this or any other phase of the work into its finer details we refer them to the new edition of Manson, which is a truly excellent production.

